# Giant Vesicles Produced with Phosphatidylcholines (PCs) and Phosphatidylethanolamines (PEs) by Water-in-Oil Inverted Emulsions

**DOI:** 10.3390/life11030223

**Published:** 2021-03-10

**Authors:** Boying Xu, Jinquan Ding, Jian Xu, Tetsuya Yomo

**Affiliations:** 1 Laboratory of Biology and Information Science, Biomedical Synthetic Biology Research Center, School of Life Sciences, East China Normal University, Shanghai 200062, China; byxu@bio.ecnu.edu.cn; 2 School of Software Engineering, East China Normal University, Shanghai 200062, China; 51194501115@stu.ecnu.edu.cn

**Keywords:** giant vesicles, water-in-oil inverted emulsions, IFC, lipid, membrane

## Abstract

(1) Background: giant vesicles (GVs) are widely employed as models for studying physicochemical properties of bio-membranes and artificial cell construction due to their similarities to natural cell membranes. Considering the critical roles of GVs, various methods have been developed to prepare them. Notably, the water-in-oil (w/o) inverted emulsion-transfer method is reported to be the most promising, owning to the relatively higher productivity and better encapsulation efficiency of biomolecules. Previously, we successfully established an improved approach to acquire detailed information of 1-Palmitoyl-2-oleoyl-sn-glycero-3-phosphocholine (POPC)-derived GVs with imaging flow cytometry (IFC); (2) Methods: we prepared GVs with different lipid compositions, including phosphatidylcholines (PCs), phosphatidylethanolamines (PEs), and PC/PE mixtures by w/o inverted emulsion methods. We comprehensively compared the yield, purity, size, and encapsulation efficiency of the resulting vesicles; (3) Results: the relatively higher productivities of GVs could be obtained from POPC, 1,2-dioleoyl-sn-glycero-3-phosphocholine (DOPC), 1,2-dilauroyl-sn-glycero-3-phosphoethanolamine (DLPE), DOPC: DLPE (7:3), and POPC: DLPE (6:4) pools. Furthermore, we also demonstrate that these GVs are stable during long term preservation in 4 °C. (4) Conclusions: our results will be useful for the analytical study of GVs and GV-based applications.

## 1. Introduction

To understand the organization and dynamics of lipid bilayers, research on giant vesicles (GVs) as an artificial cellular membrane model has gained considerable interest in recent decades [1,2,3,4,5,6]. GVs consisting of phospholipid membranes are similar to modern cellular membranes in terms of physical and chemical properties. Additionally, GVs have large dimensions of their typical cell sizes, making them suitable artificial compartments for the construction of synthetic cells [7,8] through the bottom-up approach [9], is very attractive in the synthetic biology field. Thus, the development of reliable protocols for the preparation and analysis of GVs in a designed environment is crucial for promoting the synthetic cell project to the next stage.

The importance of GVs has initiated a massive effort in developing an easy and efficient preparation method to produce vesicles from known lipid compositions [6]. The challenge lies in establishing a reproducible way to produce GVs with a considerable encapsulation efficacy for biomolecules in a desired yield and quality. To date, various techniques for GV preparations have been established, such as lipid hydration including simple film hydration [10] and gel- [11] or paper-assisted hydration [12], electro-formation [13,14], and emulsion phase transfer method [15,16,17]. Lipid hydration, as one of the most commonly used approaches, is easy to handle and only requires several common facilities and equipment, such as an evaporator and vacuum pump. However, it always requires experimental optimizations, and the resulting vesicles usually have polydisperse size distributions and low encapsulation efficiencies. Comparatively, GVs from the electro-formation method could produce uniform and uni-lamellar GVs, possessing a high reproducibility and lower polydispersity. Unfortunately, low yield, low encapsulation efficiency and limited lipid compositions are the main drawbacks of the electro-formation method. The microfluidic technique has recently been demonstrated as a state-of-art method of preparing GVs [18,19,20,21] in a designed micro-chip. Although a microfluidic chip does yield monodisperse GVs with higher encapsulation efficiencies in precise control over production, it often requires unique equipment setups and expertise in designing an appropriate chip, which is not always available to researchers [22,23,24]. Compared to the methods mentioned above, phase transfer methods such as the water-in-oil (w/o) emulsion transfer, has shown significant advantages, since it does not need specialized equipment, is of straightforward implementation, acceptable encapsulation efficiency, and high yield of uni-lamellar vesicles [15,16], although one drawback is that there is a possible presence of oil trapped within the bilayers depending on the protocols and reagent used, which might influence membrane mechanical properties [17,25,26]. Besides, this method can also produce GVs with a wide range of lipid compositions, e.g., phosphatidylcholines (PCs) and phosphatidylethanolamines (PEs), which are usually employed for constructing the artificial cell membrane [6,27,28].

As we know, PCs and PEs are major components in cell membranes and they are preferentially located in different leaflets of the bilayer: the outer layer for PCs and the inner for PEs [29]. Some studies have also suggested that the PC/PE ratio is a critical modulator of membrane integrity [30]. To date, many studies have already reported the productions of vesicles made from PC/PE lipids [6]. For example, PC or PE mixed with 10-20 mol% of a charged lipid (e.g., cardiolipin, CL; phosphatidic acid, PA; phosphatidylglycerol, PG; phosphatidylserine, PS) can form GVs through the gentle hydration method [31]; By the electro-formation method, GVs can be prepared with non-charged lipids [32] like PCs (1,2-dioleoyl-sn-glycero-3-phosphocholine, DOPC [33]; 1,2-dimyristoyl-sn-glycero-3-phosphocholine, DMPC [34] or egg yolk phosphatidylcholines, egg PC [35]); DOPC or egg PC can produce GVs by the w/o emulsion transfer method [36,37]. Morphological analysis of vesicles using electron microscopy techniques (SEM or TEM) is quite time-consuming and needs expensive machines and regular maintenances. Moreover, traditional microscopy observation sometimes does not precisely reflect the real results, e.g., size distribution and aspect ratio of particles. Finally, dynamic light scattering (DLS) can readily provide particle size and polydispersity index, but it is most suited for small and large liposomes, not micrometric ones [38]. DLS analysis does not provide visualized information of particles and usually mistakenly treats aggregated vesicles as one single particle. It is known that the flow cytometer (FCM) technology is convenient for analyzing GVs with statistically relevant measurements in large populations. More recently, imaging FCM (IFC) has been developed to analyze a large number of features based on morphology like size, shape, fluorescent intensity, circularity, and texture on a specific cell or compartment of a cell [39,40]. Our previous study established a straightforward protocol to investigate 1-Palmitoyl-2-oleoyl-sn-glycero-3-phosphocholine (POPC)-derived GVs quantitatively and qualitatively on Amnis ImageStream Mark II [15].

Continuing that effort in this study, we report the preparation of GVs with different lipid compositions such as PC, PE, and PC-PE mixtures in different ratios by the water-in-oil inverted emulsion method. Besides, we analyze and compare the yield, purity, size, and encapsulation efficiency of resulting vesicles via IFC analysis. Our work shows that PCs (POPC, DOPC, DMPC), PEs (1,2-dilauroyl-sn-glycero-3-phosphoethanolamine, DLPE; 1,2-dimyristoyl-sn-glycero-3-phosphoethanolamine, DMPE), and PC:PE mixtures (POPC: DLPE, DOPC: DLPE, and DMPC: DLPE) can be utilized to yield high production of GVs with significantly high encapsulation efficiency. Importantly, we also find that most of these GVs from w/o emulsion method show high stability over long incubation times at 4 °C. This comprehensive information would be useful in determining lipid species and conditions for liposome-based research.

## 2. Materials and Methods

### 2.1. Lipids, Calcein, Organic Solvents, and Other Reagents

All the lipids (PCs and PEs) used in preparing liposomes (Table 1) were purchased from Corden Pharma Switzerland LLC, Liestal, CH, except that DHPC (1,2-diheptanoyl-sn-glycero-3-phosphocholine), DPHPC (1,2-diphytanoyl-sn-glycero-3-phosphocholine), POPE (1-palmitoyl-2-oleoyl-sn-glycero-3-phosphoethanolamine) were from Avanti Lipids Polar, Inc., Alabaster, AL. Calcein (Solarbio, Beijing, China) was used as a fluorescent dye to mark the contents of GVs. Organic solvents, including mineral oil (liquid paraffin, d = 0.840 g/mL), were from Adamas-Beta Reagent (Shanghai, China), heavy mineral oil from Sigma–Aldrich (St. Louis, MO, USA), and chloroform and methanol to dissolve the lipids from Sinopharm Chemical Reagent (Shanghai, China). N-(2 Hydroxyethyl) piperazine-N′-2-ethanesulfonic acid (HEPES), sucrose, glucose, and KOH were purchased from Sigma–Aldrich (St. Louis, MO, USA).

### 2.2. Solubilized Lipids, Inner and Bottom Aqueous Solutions Preparation

All the PC lipids including POPC, DOPC, DMPC, DPPC (1,2-dipalmitoyl-sn-glycero-3-phosphocholine), DSPC (1,2-distearoyl-sn-glycero-3-phosphocholine), DHPC (1,2-diheptanoyl-sn-glycero-3-phosphocholine), DLPC (1,2-dilauroyl-sn-glycero-3-phosphocholine), and DPHPC (1,2-diphytanoyl-sn-glycero-3-phosphocholine) were dissolved in chloroform to make 100 mg/mL stock solutions. The stock solutions of PE, including POPE (1-palmitoyl-2-oleoyl-sn-glycero-3-phospho-ethanolamine) and DOPE (1,2-dioleoyl-sn-glycero-3-phosphoethanolamine), were mixed 50 mg/mL in chloroform. DMPE (1,2-dimyristoyl-sn-glycero-3-phosphoethanolamine), DLPE (1,2-dilauroyl-sn-glycero-3-phosphoethanolamine), DPPE (1,2-dipalmitoyl-sn-glycero-3-phospho-ethanolamine), and DSPE (1,2-distearoyl-sn-glycero-3-phosphoethanolamine) were dissolved in CHCl_3_/MeOH/ddH_2_O (65:35:8). The final stock solution concentration of DMPE, DLPE, and DPPE was 15 mg/mL, and 5 mg/mL for DSPE. The stock solutions were used to generate lipid–carrying oil solutions and filled with nitrogen for storage. The lipids POPC, DMPC, DPPC, DSPC, DLPE, DOPE, POPE, DPPE, and DSPE stock solutions were mixed with mineral oil to produce a 0.1 mg/mL lipid–oil solution. Heavy mineral oil was used to prepare 0.1 mg/mL lipid–oil solutions of DOPC, DHPC, DLPC, DPHPC, and DMPE (Table 2). Finally, the chloroform was evaporated by heating the lipid-oil solutions in an open tube at 80 °C for 30 min.

Two aqueous solutions, inner and bottom aqueous solutions, were made using ddH_2_O water. The inner aqueous solution contained 50 mM HEPES-KOH (pH 7.6), 500 mM sucrose, 0.2 mM calcein. The bottom aqueous solution was also prepared with 50 mM HEPES-KOH (pH 7.6) and 500 mM glucose to maintain the isosmotic conditions across the lipid bilayer membranes.

### 2.3. Preparation of Calcein-Stained Vesicles by the Inverted Emulsion Method

The w/o emulsion transfer method was chosen to produce GVs as previously described [15] with some minor modifications for some lipids. Calcein was added to the inner aqueous solution enabling quantitative analysis of GVs by flow cytometry. After the lipids were dissolved and the chloroform was entirely evaporated, the w/o emulsion was made by sonication using an ultrasonic processor (SONICS & MATERIALS, INC, VCX750, Newton, Connecticut, USA) on ice for 1 min (Ampl. 40%, pulse on/off 2 s/1 s) and equilibrated on ice for 10 min. Considering that lipid species used in this study have different structures and transition temperatures, the sonication conditions for the emulsion produced with those lipids were optimized, as listed in Table 2. The bottom aqueous solution was placed in a new microcentrifuge tube, and the w/o emulsion obtained in the previous step was gently poured on top of the bottom aqueous solution. After standing for 5−10 min to equilibrate the interface, the emulsion was centrifuged to form a pellet of GVs. The supernatant oil was removed and a fresh bottom aqueous solution was added to resuspend the pellet. Oil traces were removed by another centrifugation round. Finally, 100 μL of this GV suspension was prepared and immediately analyzed by IFC or stored at 4 °C for further stability analysis.

### 2.4. GV Analysis by Imaging Flow Cytometry (IFC)

Like Ishiodori et al. [15], we used the ImageStream Mark II system and INSPIRE acquisition software (Amnis/Millipore, Seattle, Washington, USA) to measure GVs based on the fluorescence intensities from encapsulated calcein. The emission from calcein (488 nm) was detected in Channel 2 with a 505−560 nm filter; bright field and side scatter (SSC) data were collected in Channel 4 and Channel 6 (785 nm), respectively. All samples were acquired with 60× magnification with a setup of low flow rate/high sensitivity. 50,000 events were recorded for each GV composition and the measurement data was analyzed by IDEAS analysis software (Amnis/Millipore, Seattle, Washington, USA). In this type of flow cytometry, internal size standard beads are run concurrently and used for daily calibration as well as real-time velocity detection and autofocus.

### 2.5. Confocal Microscopy Observation

The produced GVs were dropped (8 μL) on a microscope slide with a silicon imaging spacer (1 well, diam. × thickness 9 mm × 0.12 mm, Sigma–Aldrich, St. Louis, MO, USA), which were ultra-thin adhesive spacers peeled and stuck to slides to confine specimens without compression. The sample was then covered with a coverslip and left to stand for 10 min to allow the sediment of vesicles to travel to the bottom of the chamber by gravity. GV samples were observed with a Ti2-E inverted microscope (Nikon Ti2-E, Yokohama, Japan), and images were acquired using a confocal microscope (Nikon C2plus, Yokohama, Japan). The green fluorescence of calcein inside the GVs was excited by using a 488 nm laser with emission collected at 498–535 nm. Image analysis was performed using NIS-ELEMENTS C-ER software (Nikon, Yokohama, Japan).

### 2.6. Stability Analysis

GVs produced with POPC, DOPC, DLPE, DOPC: DLPE (7:3) mixture, and POPC: DLPE (6:4) mixture were stored at 4 °C for up to one month. We investigated the concentration and size distribution of the resulting GVs via IFC at indicated time points, 0 h, 6 h, 12 h, 24 h, 3 days, 1 week, 2 weeks, and 1 month. We also directly observed these GV samples under the confocal microscope (Nikon C2plus, Yokohama, Japan).

## 3. Results

### 3.1. Using IFC to Analyze GVs Produced by the w/o Emulsion Transfer Method

**Figure 1:**
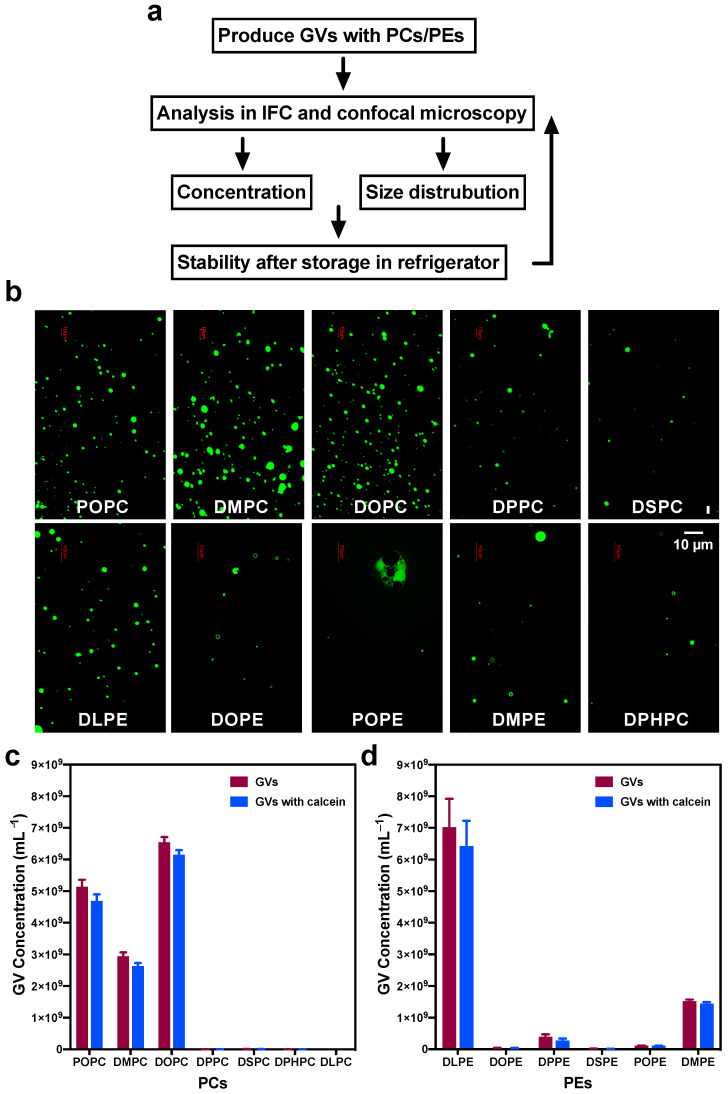
GVs produced with different phosphatidylcholines (PCs) and phosphatidylethanolamines (Pes). (**a**) The research roadmap of this study, (**b**) The fluorescence microscope images of GVs prepared with PCs and PEs. Scale bar = 10 µm. The concentration of GVs made with different PCs (**c**,**d**) PEs.

The experimental procedures are summarized in Figure 1a. An advantage of using IFC to characterize GVs was that the size distribution of all GVs could be clearly displayed and recorded. We employed IFC and microscopy to analyze the properties of GVs produced from different lipid components. As vesicles produced by the inverted w/o emulsion transfer method are of high encapsulation efficiency and mainly in the uni-lamellar form [15,16], we chose this method to prepare GVs with PCs and PEs with or without calcein encapsulations [15]. The obtained GVs were then subjected to IFC analysis as described in the “Materials and Methods” Section.

Following the procedure developed previously [15], we further made some fine-tuning for each kind of GV sample. Based on a sequence diagram for various SSC features versus brightfield, we could separate target GVs from the internal calibration beads and other byproducts such as oil droplets. Finally, we could also obtain characteristics of target GVs such as purity, calcein encapsulation efficiency, and vesicle size from the IFC analysis results as listed in Table 3 and Table S1. All the data were summarized from at least three independent replications, showing low standard deviations in each sample and outstanding reproducibility via IFC-based analysis.

### 3.2. Comparing GVs Prepared with Different PC, PE, and PC:DLPE Mixture

To investigate the effects of PC and PE species on vesicle formation, we firstly prepared GVs with seven kinds of PCs (POPC, DMPC, DOPC, DPPC, DSPC, DPHPC, and DLPC) or six kinds of PEs (DLPE, DOPE, DPPE, DSPE, POPE, and DMPE). Before IFC and confocal observation, generated GVs were viewed under a fluorescence microscope as soon as they were prepared. As shown in Figure 1b (upper panel), the fluorescence microscope showed that most PCs successfully produced GVs, except for DHPC and DLPC which yielded inferior productions (Figure S1). For GVs prepared with PEs, DLPE obtained a relatively higher yield of GVs as compared with DOPE, POPE, and DMPE species (Figure 1b, lower panel). However, the fluorescence microscope could barely detect GV products with DSPE (data not shown). The size of DLPE-derived vesicles seems smaller than DMPC-derived ones as judged from the fluorescence microscopy.

Then we investigated all the GV samples via IFC, with which we calculated the concentration of produced GVs. As plotted in Figure 1c, POPC, DMPC, and DOPC had higher yields of GVs than DPPC, DSPC, DPHPC, and DLPC. Overall, DOPC could achieve the highest vesicle production (6.54 ± 0.17 × 10^9^ vesicles/mL) and calcein encapsulation efficiency (94.12 ± 0.30%, Table 3). Out of 13 kinds of lipids tested in this study, the concentration of GVs from DLPE reaches the highest at 7.02 ± 0.90 × 10^9^ vesicles/mL (Figure 1d and Table 3). These results acquired from IFC were mainly consistent with the microscopy images. Moreover, with IFC we could compare detailed analysis among samples, and we conclude that POPC, DMPC, DOPC, and DLPE allow for efficient GV production.

**Figure 2:**
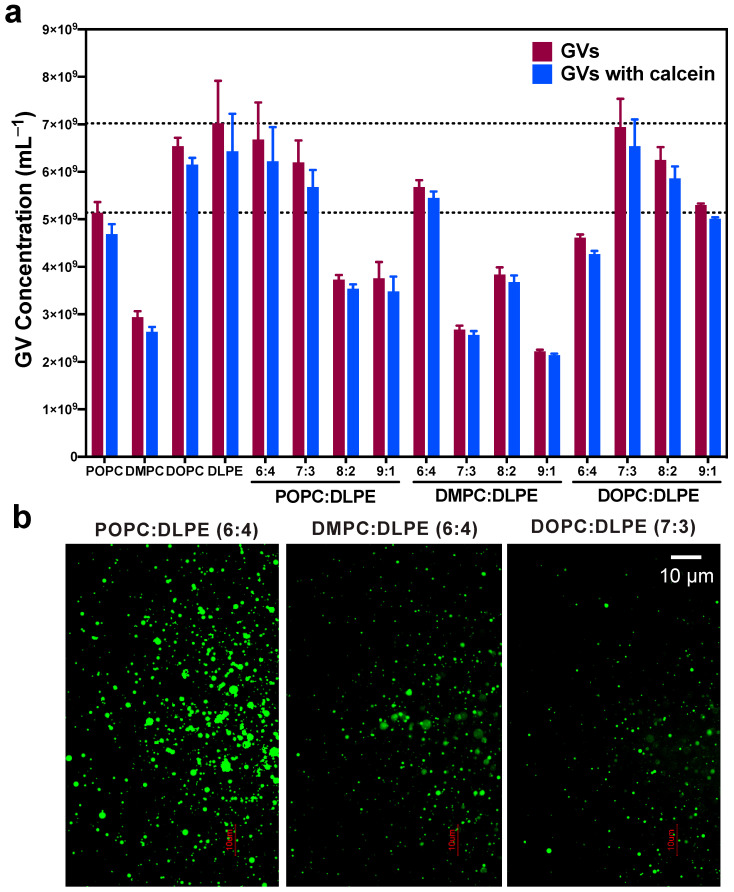
GVs produced with different ratio of PC: DLPE mixture. (**a**) The concentration of GVs prepared with POPC: DLPE, DMPC: DLPE, and DOPC: DLPE, (**b**) The fluorescence microscope images of GVs prepared with PCs: DLPE. Scale bar = 10 µm.

According to the above concentration results, we tried to prepare GVs with mixed POPC, DOPC, and DMPC with DLPE in ratios of 6:4, 7:3, 8:2, and 9:1, because it was reported that the PC/PE ratio is a critical modulator of membrane integrity [30,41]. For GVs with POPC: DLPE mixture, the yields of vesicles suggested that the extra DLPE could enhance the vesicle formation at a ratio of around 6:4 and 7:3, which is comparable to the concentration with pure DLPE (Figure 2a). We obtained the highest yield vesicle product of 6.68 ± 0.78 × 10^9^ vesicles/mL in the ratio of 6:4 (Table 3). When DOPC was mixed with DLPE in a ratio of 7:3, the mixture reached the highest yields of 6.94 ± 0.60 × 10^9^ vesicles/mL, which was a little higher than the mix in the ratio of 8:2 (Figure 2a). Once DMPC mixed with DLPE in the proportion 6:4, the concentration of produced GV (5.68 ± 0.14 × 10^9^ vesicles/mL) was obviously higher than GVs in the ratio of 7:3, 8:2, and 9:1 (Figure 2a). The fluorescence microscope showed an apparent difference in the concentration of vesicles among POPC: DLPE-, DOPC: DLPE-, and DMPC: DLPE-derived GVs (Figure 2b and Figure S2), which is in accordance with IFC results.

### 3.3. Size Distribution-Based Stability Analysis of GVs via IFC

Since IFC can measure the size of single particles, we compared size distributions among GV composition with higher yields: POPC, DOPC, DLPE, DOPC: DLPE (7:3), and POPC:DLPE (6:4). For every sample, 50,000 particles were registered regardless of the concentrations. Purity and encapsulation efficiency were defined as the ratio of GVs excluding beads and other byproducts such as oil droplets in all obtained objects and the ratio of GVs containing calcein over all GVs, respectively. As listed in Table 3, the mean sizes of POPC and DOPC vesicles were almost identical (~4.1 μm), which are slightly smaller than DMPC (~4.7 μm). Comparatively, DLPE-derived GVs are the smallest (~3.7 μm). When mixed with DLPE, PCs/DLPE also generate smaller GVs, ranging from 3.4 to 3.8 μm. As for the encapsulation efficiency, although internal calcein can leak from inside of the GVs to outside solutions, all GVs tested have ~90% or more vesicles encapsulated indicating that the w/o emulsion method is suitable for encapsulating materials inside of GVs.

**Figure 3:**
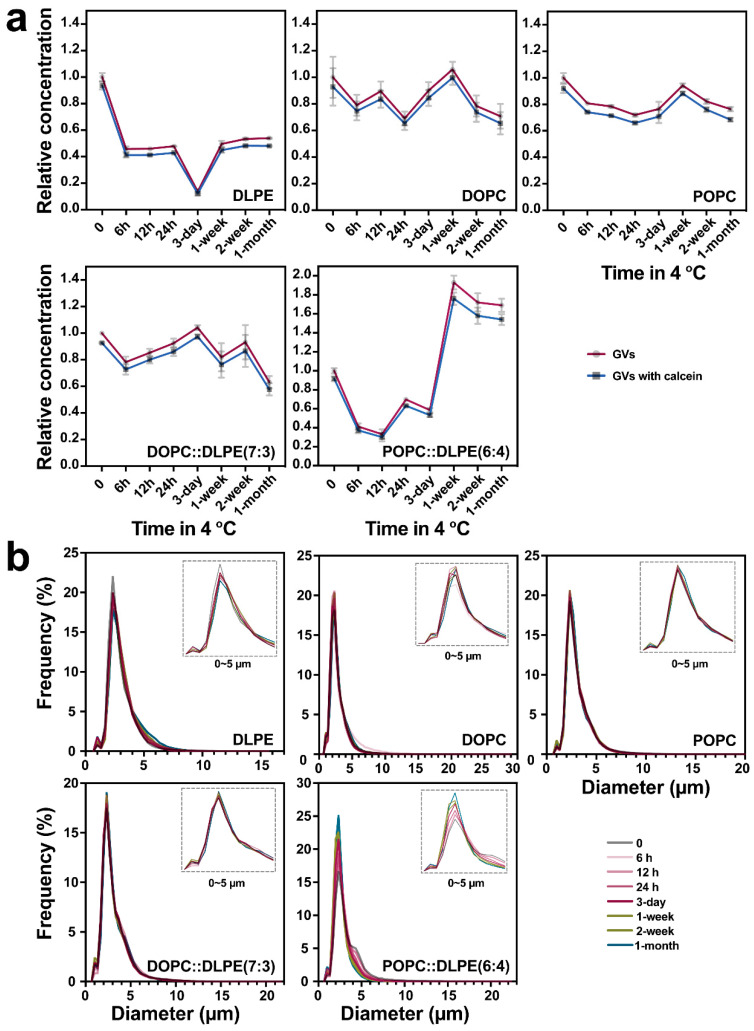
Stability analysis of GVs. The relative concentration (**a**) and the size distribution (**b**) of GVs at different time points (0, 6 h, 12 h, 24 h, 3 d, 1 week, 2 weeks, and 1 month) during storage at 4 °C.

**Figure 4:**
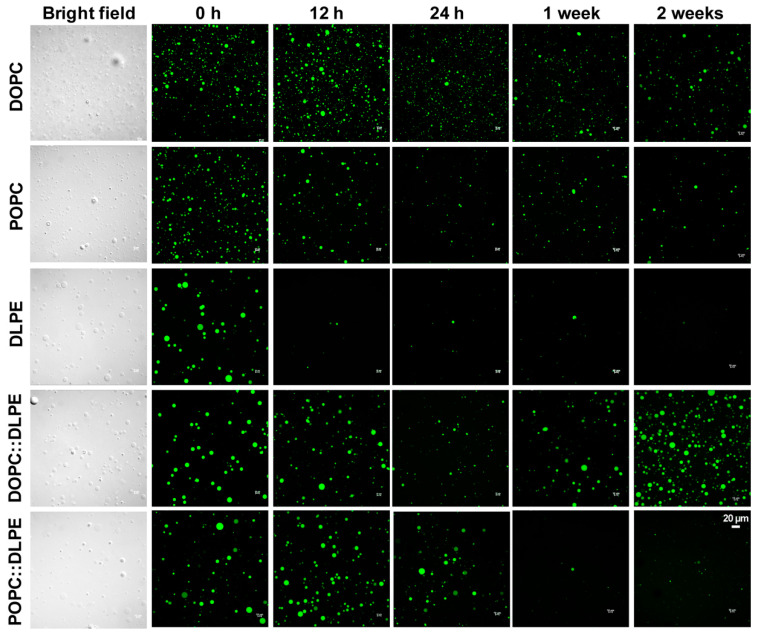
Confocal images of high yield of GVs at different time points (0 h, 12 h, 24 h, 1 week, and 2 weeks) during storage at 4 °C.

We then investigated the dynamics of the concentration (Figure 3a) and size distribution (Figure 3b) of the above GVs (with or without calcein encapsulation) stored at 4 °C for indicated time periods up to one month. IFC analysis results showed that the yield of DLPE vesicles reduced almost by half at 6 h, and the concentration was the lowest at 3 d, to ~10%, indicating the instability of DLPE-derived GVs. The overall reduction of GV concentrations with DOPC or POPC fluctuated at a range of 20% to 40%. For GVs of DOPC:DLPE (7:3), the trend of the concentration changes at different time points is similar to DOPC vesicles. For GVs of POPC:DLPE (6:4), it is interesting to observe that the yields dropped to almost 20% at 12 h. Afterward, it increased to 1.8-fold at 1 w and then started to decline again (Figure 3a). As shown in Figure 3b and Figure 4, there was an apparent increase in the population of smaller size POPC:DLPE (6:4)-derived vesicles starting from 1 week, which could be the consequence of the long-term storage at 4 °C. For the whole size distribution of GVs, most GVs produced with DLPE, DOPC, POPC, and DOPC:DLPE were almost the same during the 1-month storage (Figure 3b). Collectively, except for DLPE, GVs produced with DOPC, POPC, DOPC: DLPE (7:3), and POPC: DLPE (6:4) were stable.

To show a comparative analysis between IFC and microscopy, we further provide a series of observations under the confocal microscope, as shown in Figure 4. Basically, the fluorescence images could not give quantitive results, but rather trends for quality control. As experienced in our study, the results from microscopy images are greatly influenced by the resolution of the machine and the spot or area observed. For instance, fluorescent images showed that the yield of GVs of DOPC:DLPE (7:3) at 1 month seems higher than at 0 h (Figure 4), probably due to the heterogeneity of GVs when we prepared the glass sample. Another example is that GVs of POPC:DLPE (6:4) became smaller at 2 weeks and 1 month, but the concentrations showed almost no significant difference to the control (Figure 3a and Figure 4).

## 4. Discussion

Recently, there has been a lively interest in using GVs to build either protocells or artificial cells that mimic the biological functions of cells [4,8,42]. The broad application prospects of GVs incentivizes the development of GV preparation methods and state-of-the-art analytical approaches [6]. The w/o inverted emulsion-based process, compared with others such as gentle hydration, electro-formation or microfluidic, has benefits of high encapsulation efficiency and yield of uni-lamellar vesicles with a wide range of lipid compositions [15,27,28]. In this work, using the w/o inverted emulsion-based method, we prepared GVs with different kinds of phosphatidylcholines (PCs) and phosphatidylethanolamines (PEs), which are two major phospholipids of the plasma membrane. Once we prepared the GVs, we firstly used a fluorescence microscope to observe them. Based on microscopy results, we can only detect whether or not the GVs formed and estimate the rough concentrations.

However, following the analysis of the imaging flow cytometer, which combines high-resolution microscopy technology with traditional flow cytometry [15], we can perform extensive qualitative and quantitative imaging analysis of particles in a straightforward manner. The vesicle and non-vesicle particles, such as oil droplets or vesicle-oil mixture, and vesicles with or without encapsulations, can be divided using, via imaging, all vesicles at a single particle level. Moreover, vesicle shape changes such as budding or bursting, vesicle fusion, or fission can also be detected and analyzed accordingly, depending on the experimental design. More importantly, the concentration, the size or size distribution, and the encapsulation efficiency of GVs can be measured accurately, which increase reproducibility significantly.

It has been reported in part that PC and PE, mainly egg PC [31,35,36,37], DOPC [33,36,37,43], POPC [17,44,45], DMPC [34,46], and DPPE [46] can be used to produce GVs by gentle hydration, electro-formation, or the w/o emulsion transfer method. By the gentle hydration method, DOPC/DOPE-PEG2000 [47], POPC/DPPE-PEG2000 [44] can form GVs; e.g., PC/POPE can prepare GVs by the electro-formation method [35]. So far, there is a lack of comprehensive study aiming at producing GVs with various kinds of PC and PE lipids. Here, we have prepared GVs with various kinds of single PC, PE, and PC:PE mixtures. We demonstrate that PCs including POPC, DOPC, DMPC, PEs containing DLPE, DMPE, and POPC:DLPE mixture, DOPC:DLPE mixture, and DMPC:DLPE mixture can yield a high production of GVs. The concentration and the size distribution of these GVs are relatively stable during lengthy time storage. Considering that cylindrical PCs and cone-shaped PEs promote vesicle-vesicle hemi-fusion [48], our findings will aid in vesicle preparation research, vesicle–vesicle fusion studies, and further developments of GVs in artificial membrane and artificial cell research.

## Figures and Tables

**Table 1:** Chemical information on all lipids used to produce giant vesicles (GVs).

Lipids	CAS No.	Formula	Structure
DHPC (1,2-diheptanoyl-sn-glycero-3-phosphocholine	39036-04-9	C_22_H_44_NO_8_P	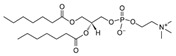
DLPC (1,2-dilauroyl-sn-glycero-3-phosphocholine	18194-25-7	C_32_H_64_NO_8_P	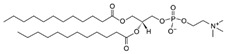
DMPC (1,2-dimyristoyl-sn-glycero-3-phosphocholine)	18194-24-6	C_36_H_72_NO_8_P	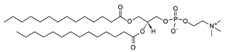
DPPC (1,2-dipalmitoyl-sn-glycero-3-phosphocholine)	63-89-8	C_40_H_80_NO_8_P	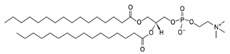
DPHPC (1,2-diphytanoyl-sn-glycero-3-phosphocholine)	207131-40-6	C_48_H_96_NO_8_P	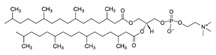
DSPC(1,2-distearoyl-sn-glycero-3-phosphocholine)	816-94-4	C_44_H_88_NO_8_P	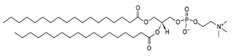
DOPC (1,2-dioleoyl-sn-glycero-3-phosphocholine)	4235-95-4	C_44_H_84_NO_8_P	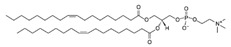
POPC (1-Palmitoyl-2-oleoyl-sn-glycero-3-phosphocholine)	26853-31-6	C_42_H_82_NO_8_P	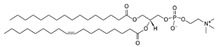
DLPE (1,2-dilauroyl-sn-glycero-3-phosphoethanolamine)	59752-57-7	C_29_H_58_NO_8_P	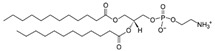
DMPE (1,2-dimyristoyl-sn-glycero-3-phosphoethanolamine)	998-07-2	C_33_H_66_NO_8_P	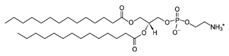
DPPE (1,2-dipalmitoyl-sn-glycero-3-phospho-ethanolamine	923-61-5	C_37_H_74_NO_8_P	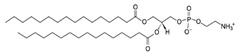
DSPE (1,2-distearoyl-sn-glycero-3-phosphoethanolamine)	1069-79-0	C_41_H_82_NO_8_P	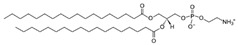
DOPE (1,2-dioleoyl-sn-glycero-3-phosphoethanolamine)	4004-05-1	C_41_H_78_NO_8_P	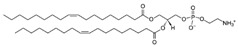
POPE (1-palmitoyl-2-oleoyl-sn-glycero-3-phosphoethanolamine)	26662-94-2	C_39_H_76_NO_8_P	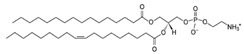

**Table 2:** Sonication conditions for emulsion produced with different lipids.

Lipids	Oil	Sonication Conditions
DHPC	hMO ^1^	sonication on ice, equilibration on ice
DLPC	hMO	sonication on ice, equilibration on ice
DMPC	MO ^2^	sonication at RT, equilibration at RT
DPPC	hMO	sonication on ice, equilibration at RT
DPHPC	hMO	sonication on ice, equilibration on ice
DSPC	MO	sonication at RT, equilibration at RT
DOPC	hMO	sonication on ice, equilibration at RT
POPC	MO	sonication on ice, equilibration on ice
DLPE	MO	sonication on ice, equilibration at RT
DMPE	hMO	sonication on ice, equilibration at RT
DPPE	hMO	sonication on ice, equilibration at RT
DSPE	MO	sonication on ice, equilibration at RT
DOPE	hMO	sonication on ice, equilibration on ice
POPE	hMO	sonication on ice, equilibration at RT
POPC: DLPE	MO	sonication on ice, equilibration on ice
DOPC: DLPE	hMO	sonication on ice, equilibration at RT
DMPC: DLPE	MO	sonication at RT, equilibration at RT

^1^ heavy mineral oil, ^2^ mineral oil.

**Table 3:** Comparison of high yield of vesicles *.

GVs	Purity (%)	Concentration (Objects/mL)	Mean Diameter (µm)	Encapsulation Efficiency (%)
POPC	92.45 ± 0.30	5.14 ± 0.22 ×10^9^	4.08 ± 0.15	91.07 ± 0.15
DOPC	96.85 ± 0.13	6.54 ± 0.17 ×10^9^	4.09 ± 0.05	94.12 ± 0.30
DMPC	93.33 ± 0.21	2.94 ± 0.13 ×10^9^	4.69 ± 0.06	89.24 ± 0.35
DLPE	97.01 ± 0.71	7.02 ± 0.90 ×10^9^	3.66 ± 0.29	91.61 ± 0.35
POPC: DLPE_6:4	97.64 ± 0.09	6.68 ± 0.78 ×10^9^	3.75 ± 0.06	93.07 ± 0.04
DOPC: DLPE_7:3	93.10 ± 0.32	6.94 ± 0.60 ×10^9^	3.76 ± 0.10	94.21 ± 0.37
DMPC: DLPE_6:4	91.61 ± 0.32	5.68 ± 0.14 ×10^9^	3.44 ± 0.02	96.03 ± 0.05

* represents means ± standard error, Sample size (n) = 3.

## Data Availability

Data is contained within the article.

## Supplementary Materials

The following are available online at https://www.mdpi.com/2075-1729/11/3/223/s1, Figure S1: The fluorescence microscope images of GVs prepared with all PCs and PEs used in this study, Figure S2: The fluorescence microscope images of GVs prepared with PCs: DLPE, Table S1: Characterization of vesicles produced with other lipids resulting lower yields.
