# Supplementary material for: Giant Vesicles Produced with Phosphatidylcholines (PCs) and Phosphatidylethanolamines (PEs) by Water-in-Oil Inverted Emulsions
Source: Life (Basel). 2021 Mar 10;11(3):223. doi: 10.3390/life11030223 (PMC7998898; doi:10.3390/life11030223)
Supplement: Supplementary file 1 [file life-11-00223-s001.zip › Fig. S2.pdf]

Fig. S2

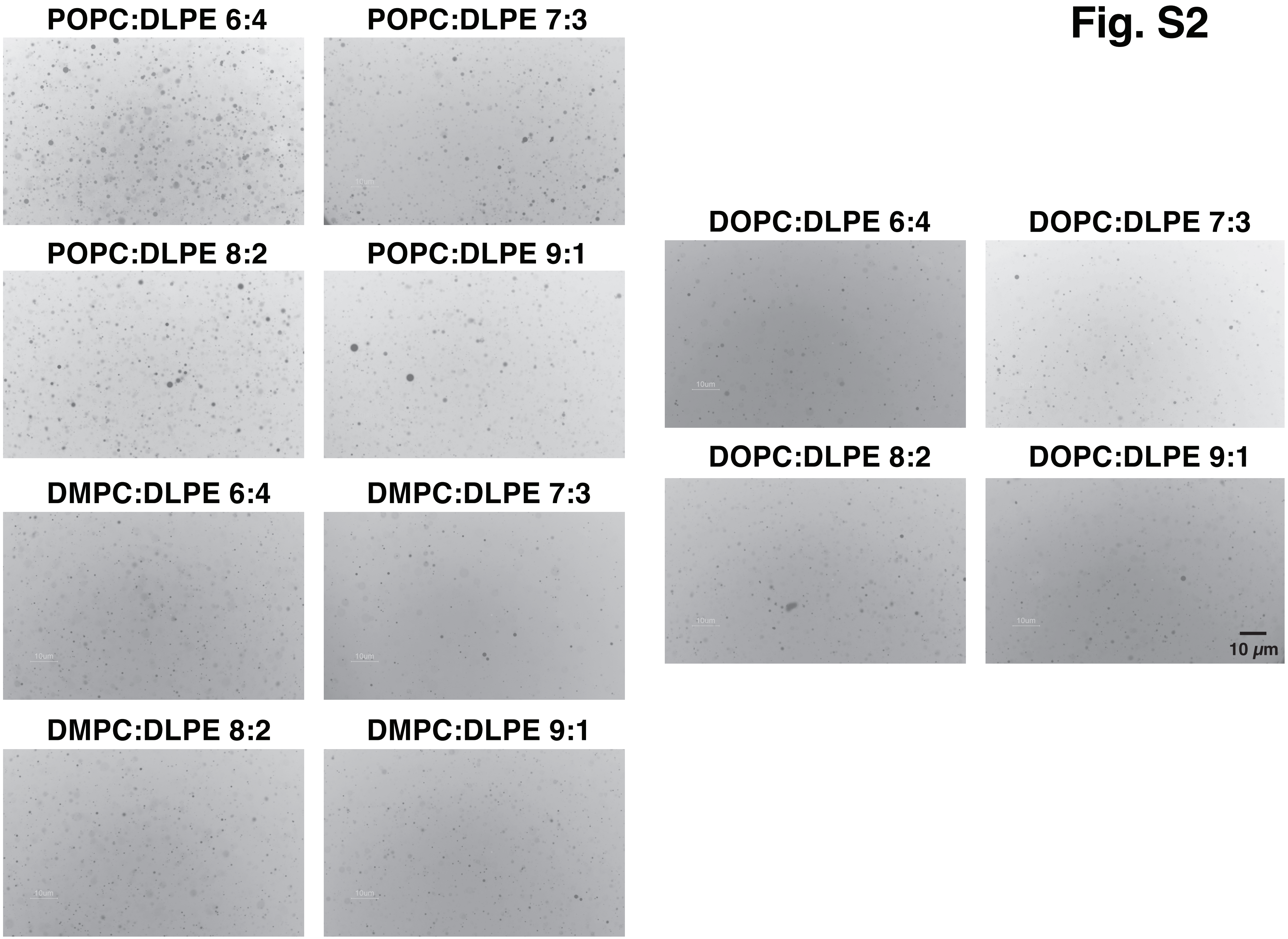
POPC:DLPE 6:4
POPC:DLPE 7:3
POPC:DLPE 8:2
POPC:DLPE 9:1
DMPC:DLPE 6:4
DMPC:DLPE 7:3
DMPC:DLPE 8:2
DMPC:DLPE 9:1
DOPC:DLPE 6:4
DOPC:DLPE 7:3
DOPC:DLPE 8:2
DOPC:DLPE 9:1
10 μm
