# Supplementary material for: Giant Vesicles Produced with Phosphatidylcholines (PCs) and Phosphatidylethanolamines (PEs) by Water-in-Oil Inverted Emulsions
Source: Life (Basel). 2021 Mar 10;11(3):223. doi: 10.3390/life11030223 (PMC7998898; doi:10.3390/life11030223)
Supplement: Supplementary file 1 [file life-11-00223-s001.zip › Table S1.docx]

**Table S1. Characterization of vesicles produced with other lipids^*^.**

| **GVs** | **purity**  **(%)** | **concentration (objects/ml)** | **mean diameter**  **(µm)** | **encapsulation efficiency (%)** | |
| --- | --- | --- | --- | --- | --- |
| DPPC | 86.91±0.24 | 4.01±0.64 E+06 | 4.00±0.01 | | 97.88±0.19 |
| DSPC | 84.20±6.50 | 1.43±0.26 E+07 | 2.97±0.69 | | 93.23±6.35 |
| DPHPC | 42.02±12.45 | 2.96±0.90 E+06 | 3.25±0.12 | | 97.76±1.47 |
| DLPC | 95.99±0.77 | 4.96±0.34 E+06 | 4.20±0.01 | | 99.48±0.14 |
| DOPE | 24.07±6.47 | 4.52±0.32 E+07 | 3.00±0.05 | | 96.30±0.61 |
| DPPE | 48.14±4.14 | 3.94±0.81 E+08 | 4.63±0.04 | | 90.56±0.37 |
| DSPE | 17.22±4.46 | 2.30±0.11 E+07 | 2.71±0.03 | | 72.24±1.47 |
| POPE | 34.59±4.08 | 1.15±0.08 E+08 | 3.13±0.04 | | 97.65±0.34 |
| DMPE | 88.55±2.22 | 1.52±0.05 E+09 | 5.03±0.04 | | 94.92±0.19 |
| POPC: DLPE_7:3 | 89.98±1.84 | 5.00±0.28 E+09 | 3.97±0.12 | | 91.72±1.01 |
| POPC: DLPE_8:2 | 82.95±0.22 | 3.05±0.08 E+09 | 4.12±0.04 | | 94.71±0.09 |
| POPC: DLPE_9:1 | 83.14±0.92 | 2.95±0.26 E+09 | 3.78±0.08 | | 92.41±0.07 |
| DOPC: DLPE_6:4 | 90.24±0.73 | 4.61±0.07 E+09 | 4.24±0.02 | | 92.77±0.09 |
| DOPC: DLPE_8:2 | 94.10±0.14 | 6.25±0.27 E+09 | 3.66±0.04 | | 93.84±0.07 |
| DOPC: DLPE_9:1 | 92.64±0.60 | 5.30±0.03 E+09 | 3.83±0.03 | | 94.55±0.06 |
| DMPC: DLPE_7:3 | 89.41±1.05 | 2.68±0.08 E+09 | 3.69±0.05 | | 95.74±0.13 |
| DMPC: DLPE_8:2 | 90.34±0.36 | 3.84±0.15 E+09 | 3.91±0.06 | | 95.92±0.08 |
| DMPC: DLPE_9:1 | 89.78±1.12 | 2.22±0.03 E+09 | 3.60±0.02 | | 96.53±0.11 |

^*^Data represent the means ± standard error, n = 3.
