# Supplementary figures and images for: Giant Vesicles Produced with Phosphatidylcholines (PCs) and Phosphatidylethanolamines (PEs) by Water-in-Oil Inverted Emulsions
Source: Life (Basel). 2021 Mar 10;11(3):223. doi: 10.3390/life11030223 (PMC7998898; doi:10.3390/life11030223)

**Fig. S1**

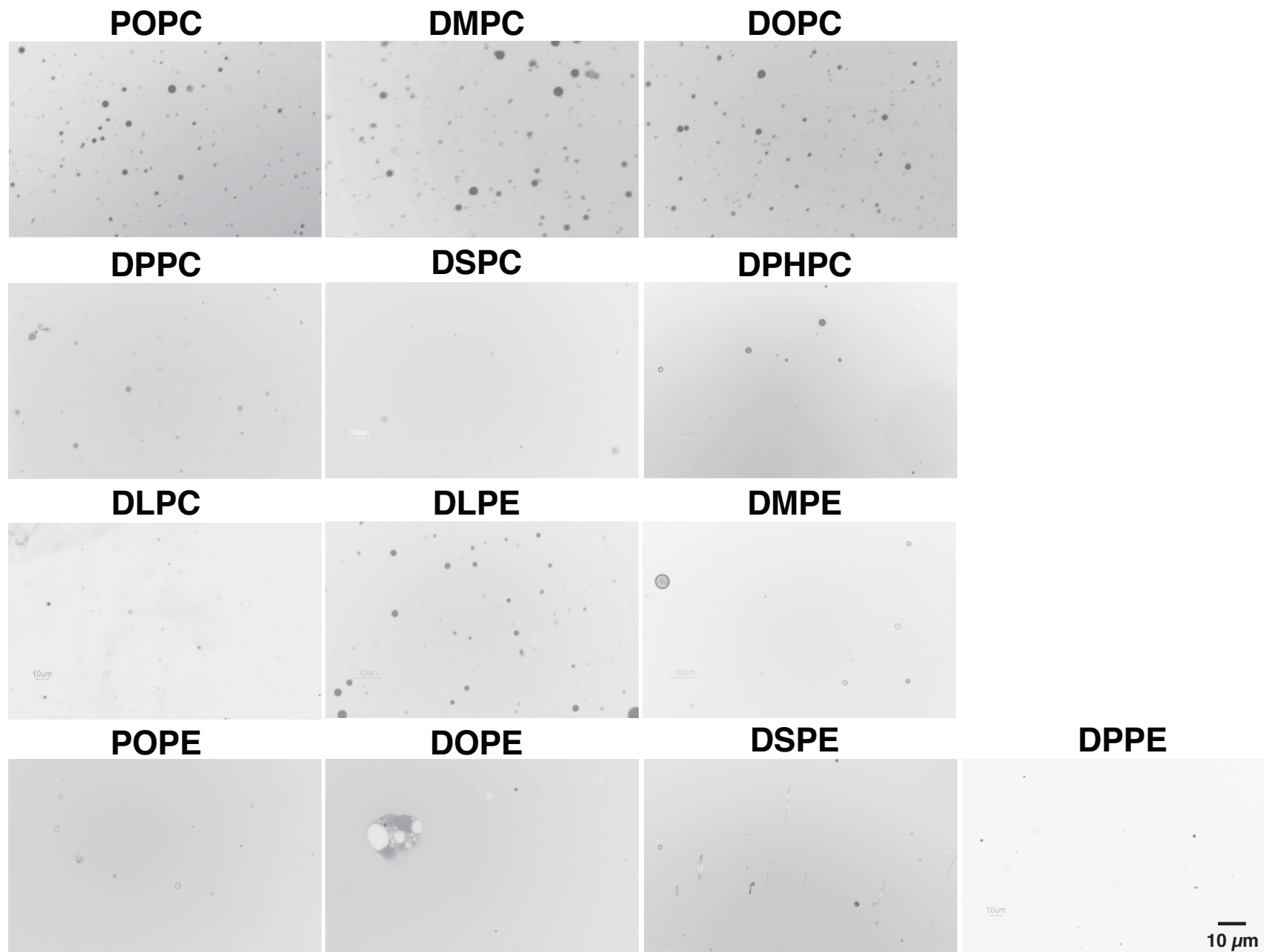

Supplement: Supplementary file 1 [file life-11-00223-s001.zip › Fig. S1.pdf]
